# Swedish guidelines for registry-based randomized clinical trials

**DOI:** 10.1080/03009734.2018.1550453

**Published:** 2019-02-06

**Authors:** Karl Nyberg, Peter Hedman

**Affiliations:** Uppsala Clinical Research Center, Uppsala University, Uppsala, Sweden

**Keywords:** Cost efficiency, healthcare registries, national guidelines, register-based randomized clinical trial, TASTE study

## Abstract

During the last decade Sweden has invested in a national infrastructure for collection of structured clinical data in the form of healthcare registries (in Sweden known as *Kvalitetsregister*). These data can be combined with other public data using the national personal identifiers that are issued to Swedish citizens. The healthcare registries have an almost complete coverage of Swedish healthcare, and a large network of clinicians is involved in the quality assurance and continuous improvement of healthcare using these registries. Uppsala Clinical Research Center (UCR) has been a technology provider of large-scale national registries and has a strong background in clinical trial management. This effort combines the areas of healthcare registries and clinical trials into a novel way of performing clinical trials to be able to: 1) run clinical trials as an integrated part of normal clinic workflow; and 2) leverage the nationwide network of outcome reporting. This strategy was shown to be successful in the TASTE (Thrombus Aspiration in Myocardial Infarction) study. When TASTE had been published, the *New England Journal of Medicine* wrote a perspective on the study calling it ‘The randomized registry trial—the next disruptive technology in clinical research?’ Since then several studies have been conducted in this way with great success. UCR has been appointed, by Clinical Studies Sweden and the Swedish Research Council, to develop the Swedish national guidelines for registry-based randomized clinical trials in order to ensure the possibility for more organizations to run this kind of study. This paper describes key concepts of register-based randomized clinical trials and the development of Swedish national guidelines.

## Registry-based randomized clinical trials—key benefits

### Introduction

A prospective trial contains several important elements that are necessary for its successful performance and completion: identification of eligible patients, obtaining consent, randomization, collection of baseline variables, endpoint detection, and endpoint adjudication. A clinical registry can be used for most of these aspects of a trial. By including the possibility of randomization in a clinical quality registry, it is possible to combine some of the critical attributes of a prospective randomized trial with the practical features of a large-scale clinical registry including the key strength of minimally selected consecutive enrollment and automated patient identification and follow-up. We describe a prospective randomized trial linked to a registry as a prospective registry-based randomized clinical trial (RRCT) by way of analogy with a prospective randomized clinical (or controlled) trial (RCT). The RRCT is an efficient and effective mechanism to assess hard clinical endpoints in large patient cohorts. Hitherto, registry-based randomized trials have evaluated treatment strategies and devices or simple pharmaceutical agents, but there is no strict limit to what therapy can be evaluated with the registry link, as long as patient safety is assured and there is adherence to existing regulations. Registry-based RCTs with more efficient and streamlined trial conduct may also be possible for evaluation of approved pharmaceutical agents for new indications and labels.

The benefit of the RRCT is related to the ability to identify patients, enroll larger numbers of patients in relatively short time, and a possibility of indefinite follow-up. This together with the fact that an RRCT often is much more inexpensive than an ordinary RCT has made the RRCT renowned on a global scale.

### Evidence-based selection of inclusion criteria

The registry data collections provide a unique reference set when deciding on suitable inclusion criteria for the clinical trial. Statisticians work closely with investigators to infer the optimal balance between population size and event rates based on the large body of historical data in the registries.

### In-registry enrollment of patients

Enrollment of patients to the study can be done in the registry application. This inclusion can be based on reported patient demographics and medical background available in the registry, like age, sex, comorbidity, previous trial participation, etc.

The inclusion can also be extended with trial-specific data that form a prerequisite for the enrollment of e.g. informed consent and specific laboratory analyses.

### In-registry randomization to strategy

Randomization to a treatment strategy can be done in the registry for RRCTs. This is typically done either prior to or during an intervention.

In-registry randomization can be achieved when the registry is used for reporting prior to the treatment strategy selection, for instance during an intervention in the operating room.

The randomization can be stratified (e.g. by site) and be tailored to support any number of strategies.

### Distributed clinical outcome reporting

Endpoints and specified events can be designed to be automatically collected from the normal reporting workflow in the registry. Given the nationwide adoption of the registries, this means that any site can provide outcome reporting for a trial participant as a part of routine follow-ups following the procedure.

A common endpoint like death is required to be reported to the population registry within 48 h, and this can be automatically added to the trial data set to have a current status on safety and lost-to-follow-up.

In the case where the data normally captured during follow-ups are not enough for the trial objective, it is possible to add clinical trial-specific question panels to the registry follow-up forms.

### Long-term follow-up

Sometimes the trial needs to be continued outside the temporal scope of workflow in the registry. This can be achieved by feeding a separate eCRF with the clinical trial data model data and use this system for the long-term follow-up.

Given that the participant is properly identified in the study data model, it is possible to interlink the study database with the registry or other sources at a later stage to add data to the study.

### Continuous adjudication of clinical events

As clinical outcome reporting is distributed and happens in conjunction with the event, adjudication of the event can be done continuously during the trial. This means that the trial duration can be shortened, as the adjudication is performed while the trial progresses. It also means that a data and safety monitoring board report can be kept current and validated during the clinical trial.

Any specified event that has to be adjudicated can be automatically sent to a clinical events committee (CEC) group along with the personal identifier, site identifier, date, and other required information. The CEC group can then handle the collection of data and adjudication in their normal workflow.

## Registry-based randomized clinical trial framework

Uppsala Clinical Research Center (UCR) has developed a framework for registry clinical trials as an initiative to standardize and facilitate implementation of clinical trials in registries.

The framework specifies the domain of clinical trials and defines business rules for legal compliance. The building blocks of the framework are combined to support the clinical trial at hand and coupled to the registry after being validated against the study protocol. The clinical trial implementation is treated as a stand-alone application which shares life-cycle with the registry. This architecture handles the incompatible legal requirements between clinical trials and registries with regard to patient/participant opt-out.

It is important to note that the framework is not the same as a product and that there is a need for systems developers to implement the framework with the registry.

### Clinical trial data model

At the heart of the RCT is the clinical trial data model. This is an extension of the traditional clinical report form. The clinical trial data model is a specification of the variables that are required for the inclusion and exclusion of patients as well as the ones needed for analysis of the primary and secondary endpoints.

The clinical trial data model is:

- Defined in the study protocol
- A basis for the data management plan
- A part of the ethical approval application

This means that the data model should contain all data that are required to analyze the outcome of the trial. This in turn means that the study data set can be handled separately from the registry data to provide support for the different legal requirements on registries and clinical trials.

Any change to the study database that handles the clinical data set defined by the clinical data model is fully audited.

### Electronic data capture as an implementation of the data model

The study can be performed using an electronic data capture system (EDC) as the backend for the RRCT framework. The clinical trial model is then implemented as eCRFs in the EDC. The framework provides a seamless data transfer from the registry to the EDC, which has built-in functions for data quality monitoring and on-line reporting to continuously monitor the integrity of the data.

This architecture also allows for hybrid studies where some of the sites provide data through the registry and some through the eCRFs in the EDC.

### Clinical trial criteria

A number of criteria can be configured for key features in the study.

*Inclusion of patients*. The framework provides the formal requirements for defining inclusion criteria for patients.

*Opening and closing a trial*. The framework provides support for defining criteria for the conditions where the trial accepts inclusion of participants. This can for instance be a time interval, a maximum number of participants, or based on available stratification by site.

*Patient opt-out*. The framework supports participant withdrawal from the trial. This means that the record for the participant cannot accept new data once the opt-out has been reported. This requirement is built into the data model handler in the framework.

*Lost-to-follow-up*. In the event that a participant cannot be contacted for follow-up, the participant might have to be marked for exclusion from the trial. This requirement is built into the data model handler in the framework.

### Randomization service

The framework provides support for a randomization service that randomizes participants to a treatment strategy. This includes configurations like stratification by site and sex. The stratification and randomization can be pre-compiled for the study.

### Data capture

Two different strategies are currently employed for data capture.

*Event listeners*. The framework defines event listeners that can be configured to eavesdrop on the information flow in the registry. This generates events that are sent to the clinical trial data model. This can be used for endpoints that are designed to use existing variables in the registry.

*In-registry trial panels*. In-registry trial panels can be used to add trial-specific data capture to existing registry forms. This can also be used to add branding to the clinical trial, enroll a participant, and provide the possibility for the user to randomize a participant to a treatment strategy.

### Reporting

The framework has an export function to export the study data at the end of the study. It is also possible to review the screening log during the study.

## Swedish national guidelines

In 2017 Uppsala Clinical Research Center was appointed by Swedish Clinical Studies and the Swedish Research Council to develop Sweden’s national guidelines for RRCTs.

The purpose was to use existing knowledge and technology in order to spread the use of RRCTs and to give everyone a head start instead of having to invent processes and technology themselves. The objective is to have the guidelines publicly available for everyone who wants to run a RRCT.

The guidelines consist of documentation on how the process works and how it differs from a RCT. It also contains the different roles involved and when one should, and should not, use RRCT. The guidelines also contain the actual technical framework together with documentation on how it works and how to implement it.

The guidelines are available to use and adapt to a specific study and technical environment. The guidelines can be used freely, but appropriate credit must always be given. (The national guidelines for randomized registry-based trials are available from the regional node for clinical studies or the regional office of the national registry center.)

## Case study: Thrombus Aspiration in Myocardial Infarction (TASTE)

TASTE study details are given in Table 1.

**Table 1.** Thrombus aspiration in myocardial infarction (TASTE).

TASTE study details	
Enrollment	7243
Study start date	July 2010
Study completion date	August 2013
Primary completion date	March 2013
ClinicalTrials.gov	NCT01093404

### Objective

Treatment of myocardial infarction (blood clot in the arteries of the heart) has improved after introduction of 24/7 balloon angioplasty to open the blocked artery. The clot itself is not routinely removed, but recent data in smaller trials indicate that this might improve recovery and prognosis. In this multicenter study of 7000 patients referred to Scandinavian hospitals for myocardial infarction, the investigators test the hypothesis that patients randomized to treatment with thrombus aspiration (removing the blood clot by manual suction) before conventional angioplasty will have a reduced risk of death as the primary outcome, and fewer rehospitalizations, fewer new myocardial infarctions, reduced risk of heart failure, better coronary artery flow after angioplasty, and greater reduction of infarct size compared to patients randomized to conventional angioplasty alone.

The study was run using the SWEDEHEART registry from 27 sites in Sweden, Iceland, and Denmark (1,2). The registry helped identifying patients suitable for inclusion by selecting those with a Swedish personal identification number above the age of 18 with STEMI, registered with an indication for PCI, and no previous enrollment. When the system proposed enrollment, the treating physician only had to check a few exclusion criteria and get verbal consent from the patient. The ethics committee requested a written confirmation of the verbal consent as soon as possible after the procedure. Randomization was performed directly in the registry clinical report form. No additional study variables were required for follow-up of study endpoints. All endpoints were automatically collected from national registries.

### Technical implementation

To support the TASTE study SWEDEHEART was complemented with the possibility to randomize the treatment strategy and to enroll participants. Randomization was stratified by site. Addition of study sites was performed by making the randomizations available to the sites. Endpoints were inferred from the variables in the registry as a part of the study analysis.
